# Anti-Müllerian hormone may regulate the number of calbindin-positive neurons in the sexually dimorphic nucleus of the preoptic area of male mice

**DOI:** 10.1186/2042-6410-4-18

**Published:** 2013-10-11

**Authors:** Walter Wittmann, Ian S McLennan

**Affiliations:** 1 Brain Health Research Centre, Department of Anatomy, University of Otago, PO Box 913, Dunedin 9054, New Zealand; 2 Current address: Umeå Center for Molecular Medicine, Umeå University, Umeå SE-901 87, Sweden

**Keywords:** Sexual dimorphic nucleus, Anti-Müllerian hormone, Puberty, Development, Childhood, Calbindin, Medial preoptic area, Imprinting

## Abstract

**Background:**

The male brain is putatively organised early in development by testosterone, with the sexually dimorphic nucleus of the medial preoptic area (SDN) a main exemplifier of this. However, pubescent neurogenesis occurs in the rat SDN, and the immature testes secrete anti-Müllerian hormone (AMH) as well as testosterone. We have therefore re-examined the development of the murine SDN to determine whether it is influenced by AMH and/or whether the number of calbindin-positive (calbindin^+ve^) neurons in it changes after pre-pubescent development.

**Methods:**

In mice, the SDN nucleus is defined by calbindin^+ve^ neurons (CALB-SDN). The number and size of the neurons in the CALB-SDN of male and female AMH null mutant (*Amh*^*-/-*^) mice and their wild-type littermates (*Amh*^*+/+*^) were studied using stereological techniques. Groups of mice were examined immediately before the onset of puberty (20 days postnatal) and at adulthood (129–147 days old).

**Results:**

The wild-type pre-pubertal male mice had 47% more calbindin^+ve^ neurons in the CALB-SDN than their female wild-type littermates. This sex difference was entirely absent in *Amh*^*-/-*^ mice. In adults, the extent of sexual dimorphism almost doubled due to a net reduction in the number and size of calbindin^+ve^ neurons in females and a net increase in neuron number in males. These changes occurred to a similar extent in the *Amh*^*-/-*^ and *Amh*^*+/+*^ mice. Consequently, the number of calbindin^+ve^ neurons in *Amh*^*-/-*^ adult male mice was intermediate between *Amh*^*+/+*^ males and *Amh*^*+/+*^ females. The sex difference in the size of the neurons was predominantly generated by a female-specific atrophy after 20 days, independent of AMH.

**Conclusions:**

The establishment of dimorphic cell number in the CALB-SDN of mice is biphasic, with each phase being subject to different regulation. The second phase of dimorphism is not dependent on the first phase having occurred as it was present in the *Amh*^*-/-*^ male mice that have female-like numbers of calbindin^+ve^ neurons at 20 days. These observations extend emerging evidence that the organisation of highly dimorphic neuronal networks changes during puberty or afterwards. They also raise the possibility that cellular events attributed to the imprinting effects of testosterone are mediated by AMH.

## Background

The sexually dimorphic structures in the brain are generally considered to form early in development, with the role of pubescent hormones limited to activation and refinement of existing neuronal networks [1-3]. However, this concept dates from an era when remodelling of the postnatal brain was thought impossible. Consequently, most historic experiments relating to the control of neuron number have endpoints before puberty and would therefore not detect a pubescent change in cell number. Pubescent neurogenesis [4] and pubescent loss of cortical neurons [5] have recently been reported in rodents, creating a rationale to reassess when cell number is established in the highly dimorphic brain nuclei. We report here that the number of calbindin-positive (calbindin^+ve^) neurons in the sexually dimorphic nucleus of the medial preoptic area (CALB-SDN) of mice is dimorphic before puberty but with the extent of dimorphism subsequently doubling during or after puberty.

The immature testes produce anti-Müllerian hormone (AMH, also known as MIS) as well as testosterone. The role of AMH as a regulator of sexual differentiation is also changing. Historically, it was thought to have a single function, to initiate the regression of the uterus in male embryos [6]. However, AMH and its unique type 2 receptor are highly conserved genes and predate the evolution of the uterus [7]. In some teleost species (a class of ray-finned fish), AMH appears to determine sex [7,8]. These observations raise the possibility that AMH has a broader role in virilisation than previously suspected.

Contemporary studies of mice point to the brain as a site of gonadal AMH action. The immature testes of humans, rodents and other mammals continuously secrete high levels of AMH during embryonic and postnatal development until puberty, at which stage AMH levels in males decline rapidly. In men and adult male mice, plasma AMH levels are typically less than 5% of the levels during development [6,9-11]. Ovarian production of AMH, in contrast, begins as females enter puberty, with the consequence that young male and female adults have similar levels of AMH [11-13]. The AMH in blood is entirely derived from the gonads [10,14]. AMH receptors are ubiquitously expressed in developing murine neurons [14], with AMH being required for the male exploratory behaviour [14,15] and the male bias in the number of cerebellar Purkinje and spinal motor neurons [14,16,17]. The spinal bulbocavernosus neurons, in contrast, develop independently of AMH [14]. We report here that the initial sexual dimorphism in the number of neurons in the CALB-SDN is absent in *Amh*^*-/-*^ mice.

## Methods

### Animals

C57BL/6 *Amh*^-/-^ and *Amh*^+/+^ mice were littermates generated from *Amh*^+/-^ parents [18,19] (The Jackson Laboratory, Bar Harbor, ME, USA) and were housed as previously described [20]. The Animal Ethics Committee of the University of Otago approved all experiments.

Age-matched pre-pubertal (20 days old) and adult (129–147 days old) mice of both sexes were used in this study, with the adult female mice collected at a random stage of the estrous cycle. Six mice were analysed for each group. The mice were anaesthetized with ketamine (225 mg/kg) and Domitor (3 mg/kg) and transcardially perfused with 4% paraformaldehyde in 0.1 M phosphate buffer (PB), pH 7.4, using a peristaltic pump (adults, 13.3 ml/min; 20 days, 10.0 ml/min) over 3 min. The brains were incubated in the same fixative for 2 h at room temperature (RT), followed by 36 h in 4% paraformaldehyde containing 15% sucrose at 4°C and then overnight at 4°C in 30% sucrose in 0.01 M PB, pH 7.4. They were then embedded in Tissue-Tek (Sakura Finetek USA, Torrance, CA, USA), frozen on dry ice and stored at -80°C. A notch was made in the cortex of the right-hand side to enable a consistent side of the brain to be studied.

### Immunohistochemistry

The brain region containing the CALB-SDN was serially sectioned at a thickness of 20 μm in a cryostat, and every third section was immediately stained with an anti-calbindin antibody (C9848, Sigma Aldrich, St. Louis, MO, USA). The sections were stained by free-floating immunohistochemistry. Each section was incubated in a 30 mM sodium citrate buffer, pH 8.7, for 10 min at 80°C using a water bath to heat the incubation chamber. The sections were then cooled at RT for 20 min, washed twice with 0.1 M glycine in PB (0.01 M PB, pH 7.2) for 5 min and then blocked in 5% donkey serum (Jackson ImmunoResearch, West Grove, PA, USA) for 1 h at RT. The sections were then incubated in 1.85 μg/ml of mouse monoclonal anti-calbindin-D28k for 18 h at 4°C with gentle vibration. Other sections were incubated in control IgG. The sections were washed six times in washing buffer (0.01 M PB, pH 7.2, 2% NaCl, 0.1% Tween-20) for 5 min, incubated in 2 μg/ml of biotinylated donkey anti-mouse IgG (Jackson ImmunoResearch) for 1.5 h at RT and washed three times for 5 min, followed by an incubation in 1% H_2_O_2_ for 10 min to inactivate endogenous peroxidases. Sections were then washed three times in PB for 5 min, incubated with biotin/peroxidase-conjugated streptavidin amplification complex (1:200, Amersham Biosciences/GE Healthcare Ltd, Auckland, New Zealand) for 1.5 h at RT, washed three times in PB for 5 min and once in 0.1 M acetate buffer, pH 5.2, for 5 min, stained with AEC (3-amino-9-ethylcarbazole, Sigma-Aldrich) substrate for 12 min and mounted on gelatine-coated microscopy slides.

### Anatomy

**Figure 1:**
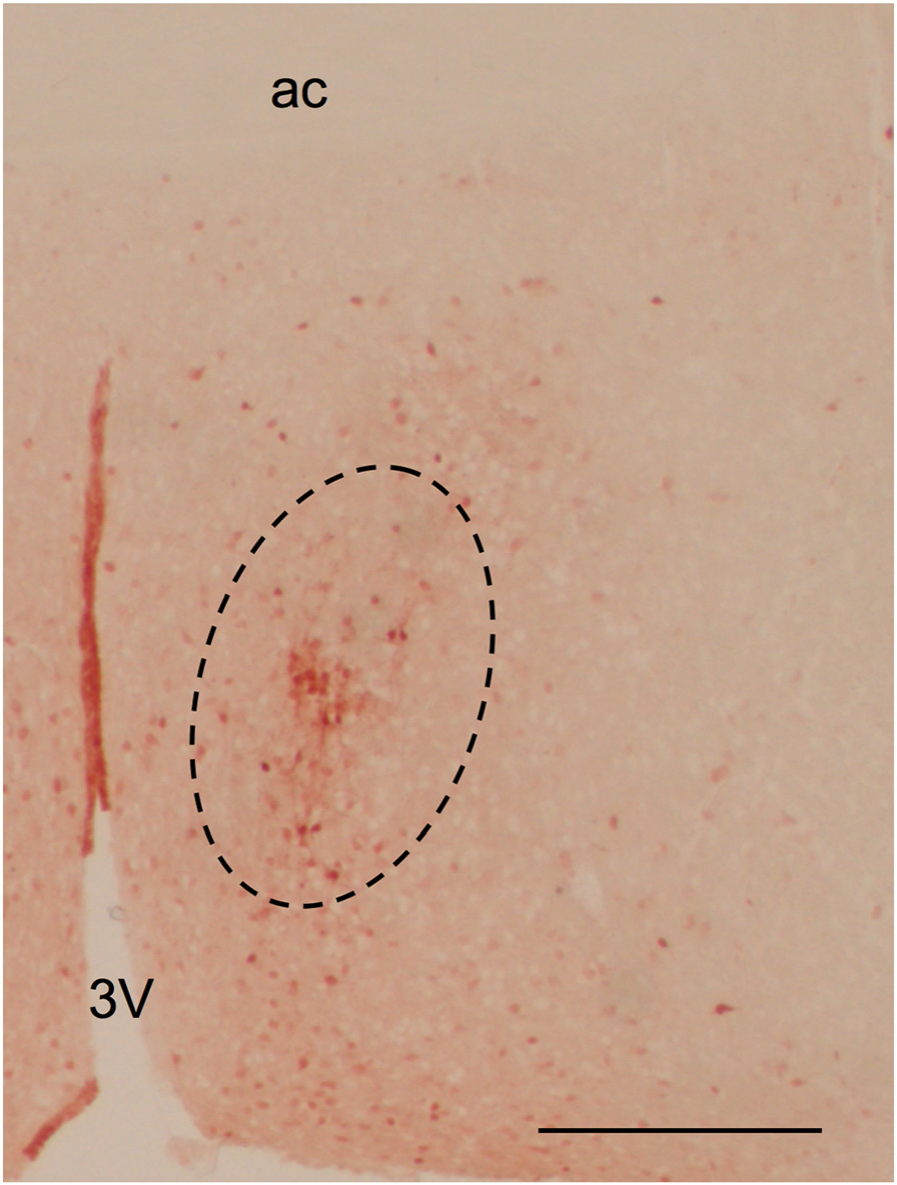
**The sexually dimorphic nucleus of the medial preoptic area.** A cryosection of an adult *Amh*^*+/+*^ male mouse was stained with an antibody to calbindin. The CALB-SDN (*dashed line*) is an ellipsoid-shaped cell cluster close to the third ventricle (*3V*) extending in a dorsolateral direction to the anterior commissure (*ac*). The *scale bar* represents 500 μm.

The sexually dimorphic nucleus of the medial preoptic area was identified by its calbindin^+ve^ neurons (Figure 1). These neurons closely overlap the sexually dimorphic nucleus identified by Nissl staining in rats [21]. The calbindin^+ve^ cluster was ellipsoidal and located 200–300 μm lateral to the third ventricle extending in the dorsolateral direction to the anterior commissure (Figure 1). This distinct cluster extended between 120 and 180 μm in the rostrocaudal direction through the medial preoptic area. The principal nucleus of the bed nucleus of the stria terminalis is also detectable by calbindin staining. Its most caudal extension can appear close to the most dorsal part of the CALB-SDN cluster, depending on the angle of sectioning. Thus, special attention was taken to only include calbindin^+ve^ cells belonging to the CALB-SDN cluster for this study. The identification and naming of the nuclei was according to Paxinos and Franklin [22].

### Stereology

The number of calbindin^+ve^ neurons in both hemispheres of each mouse was estimated using the principles of the fractionator method [23]. The entire CALB-SDN was examined on each section with a 0.75 numerical aperture lens on a light microscope (Zeiss Axioplan, Oberkochen, Germany). To ensure that all calbindin^+ve^ neurons in the CALB-SDN were examined and to avoid double counting errors, the position of each calbindin^+ve^ neuron was traced using a camera lucida. Calbindin^+ve^ cells were excluded from data collection when the perikaryon was cut at the upper plane and included when intact or cut at the lower plane of the sections. Every third consecutive section was used for calbindin staining and cell counting. The total number of calbindin^+ve^ neurons in the CALB-SDN was estimated by multiplying the number of cells counted by three.

The sizes of the perikaryon of the calbindin^+ve^ neurons were measured using the direct moment estimation of the volume of particles, which allows the unbiased estimation of volume from single sections. The diameters of neurons were measured in a random direction through a random test-point with a 0.75 numerical aperture lens using the camera lucida on a light microscope (Zeiss Axioplan). The volumes were calculated with the estimator *π*/3 times the diameter length *l*_0_ raised to the third power [23]. Approximately 20 cells per hemisphere of each animal were used for the volumetric analysis and the mean size calculated for each mouse.

The microscope slides were coded to blind the observer (WW) to the sex and genotype of the mice. All presented data are from the left hemisphere as no differences were detected between the left and right hemispheres.

### Statistics

Statistical calculations were undertaken with either PASWstatistics18.0 (SPSS Inc, IBM, Armonk, NY, USA) or GraphPad Prism software (http://www.graphpad.com). Data were examined by two-way analysis of variance (ANOVA) for (1) sex and genotype differences and (2) age and genotype differences. Significant effects were confirmed by Student’s *t* test, with *p* values of <0.05 recorded in the figures and tables.

## Results

**Figure 2:**
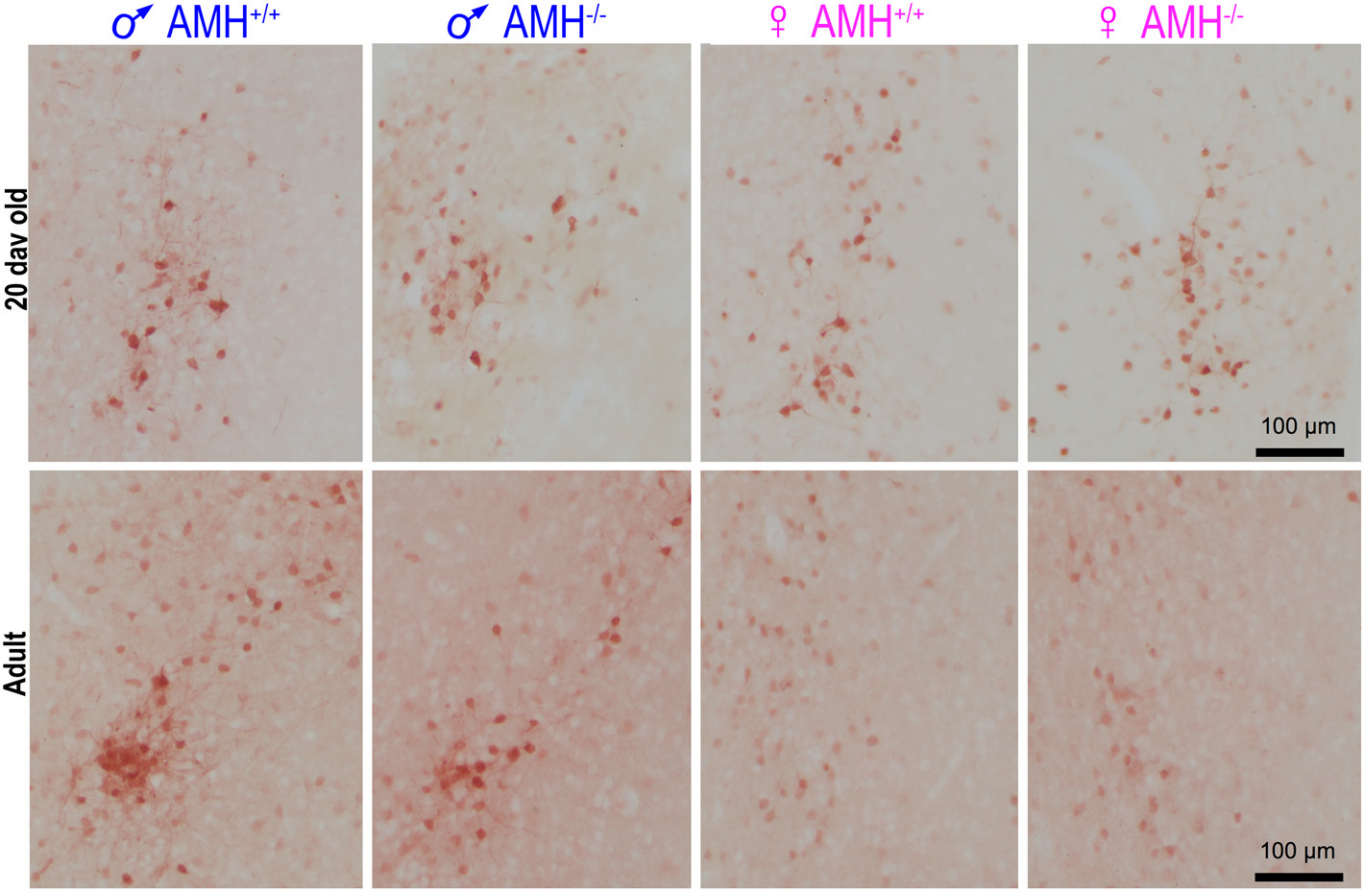
**The dimorphism in the CALB-SDN varies with age and *Amh *genotype.** The images are photomicrographs of the CALB-SDN illustrating the appearance of the nucleus in pre-pubescent (20 days old) and adult mice. All sections were stained with anti-calbindin antibodies. The location of the CALB-SDN relative to the anterior commissure and third ventricle is illustrated in Figure 1, with the quantitative estimates of the number and size of the calbindin^+ve^ neurons illustrated in Figures 3 and 4, respectively. ♂, male; ♀, female.

**Figure 3:**
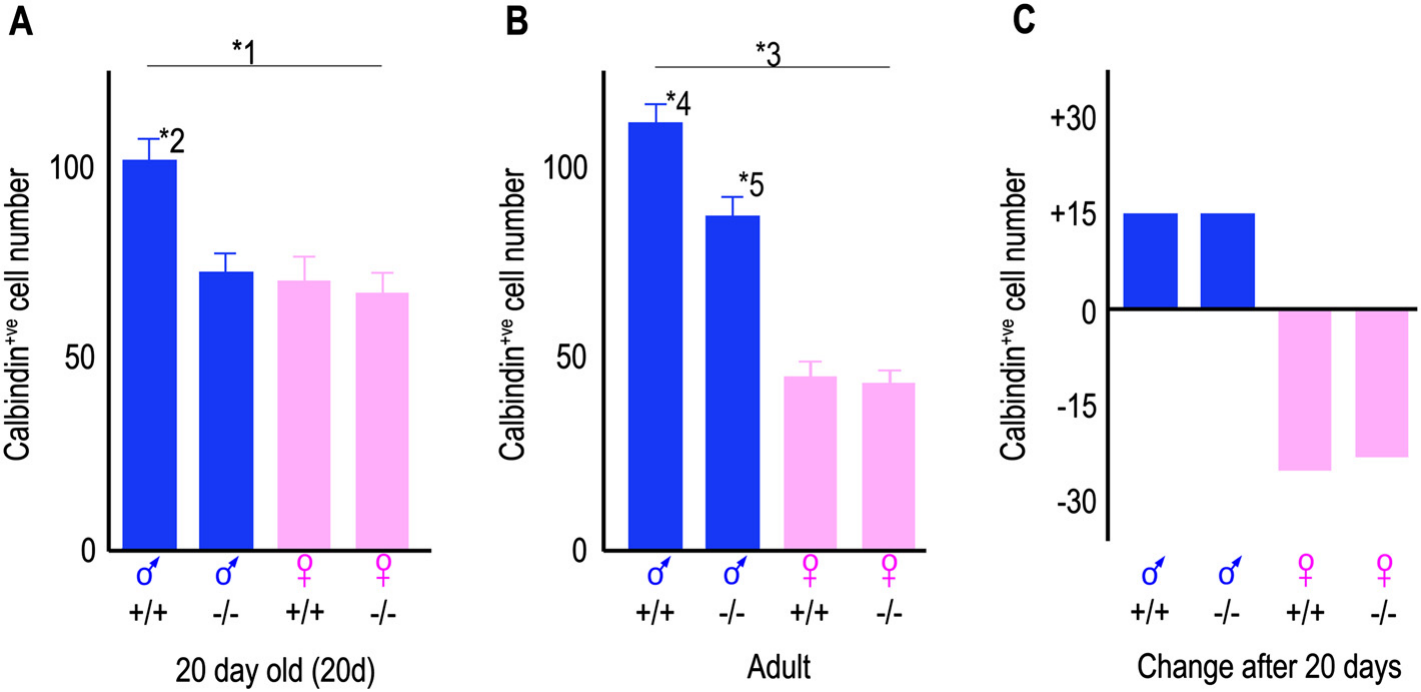
**The CALB-SDN has pre-pubescent and adult forms that are differentially regulated.** The *bars* are the mean number of calbindin^+ve^ neurons ± the standard error of the mean of six mice. The *Amh* genotype (^+/+^ or ^-/-^) is shown beneath each *bar*. **(A)** Twenty-day-old mice. *1: There was a significant effect of sex (*p* = 0.001), genotype (*p* = 0.008) and sex × genotype interaction (*p* = 0.022, two-way ANOVA). *2: The *Amh*^+/+^ males were significantly different to *Amh*^-/-^ males (*p* = 0.004) and both female groups (*p* < 0.002 *Amh*^+/+^, *p* < 0.001 *Amh*^-/-^, Student’s *t* test). **(B)** Adult mice. *3: There was a significant effect of sex (*p* < 0.001), genotype (*p* = 0.002) and sex × genotype interaction (*p* = 0.003, two-way ANOVA). *4: The *Amh*^+/+^ males were significantly different to all other adult groups (*p* = 0.002 to the *Amh*^-/-^ males and *p* < 0.001 to both female groups, Student’s *t* test). *5: The adult *Amh*^-/-^ males were also significantly different to both of the adult female groups (*p* < 0.001 *Amh*^+/+^, *p* < 0.001 *Amh*^-/-^, Student’s *t* test). **(C)** The *bars* illustrate the mean change in cell number after 20 days. In two-way ANOVAs of age and genotype, there was a significant effect of age (*p* = 0.007) and genotype (*p* < 0.001) for the male mice and a significant effect of age (*p* < 0.001) for the female mice. The adult mice were significantly different to the corresponding 20-day-old mice (*p* = 0.037 for the *Amh*^+/+^ males, *p* = 0.035 for the *Amh*^+/+^ females and *p* = 0.017 for the *Amh*^-/-^ females, Student’s *t* test). *♂*, male; ♀, female.

**Figure 4:**
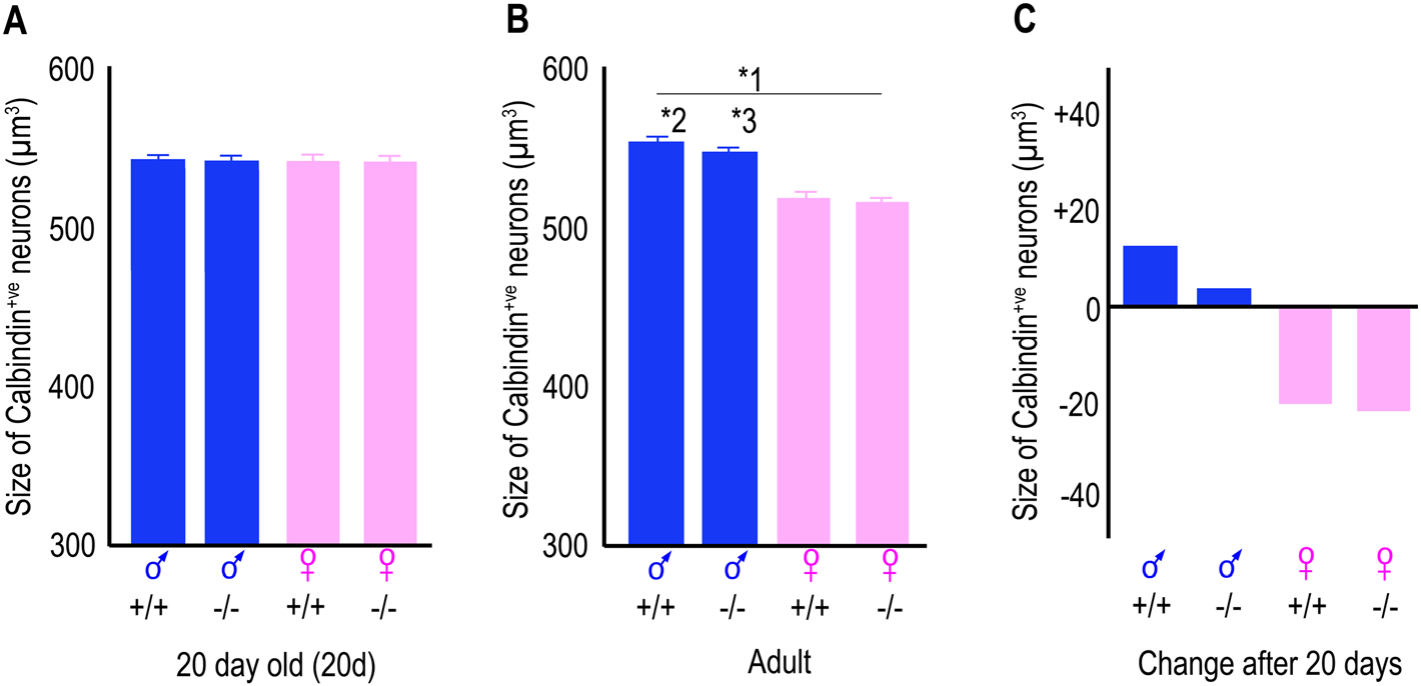
**The size of neuronal soma in the CALB-SDN is dimorphic after puberty.** The *bars* are the mean size of the cell body of the calbindin^+ve^ neurons ± the standard error of the mean of six mice. The *Amh* genotype (^+/+^ or ^-/-^) is shown beneath each *bar*. **(A)** Twenty-day-old mice. There is no effect of sex or genotype at this age. **(B)** Adult mice. *1: There was a significant effect of sex (*p* < 0.001, two-way ANOVA) but not of genotype. *2: The *Amh*^+/+^ males were significantly different to both of the female groups (*p* = 0.005 *Amh*^+/+^ and *p* = 0.004 *Amh*^-/-^, Student’s *t* test). *3: The adult *Amh*^-/-^ males were also significantly different to both of the adult female groups (*p* = 0.011 *Amh*^+/+^, *p* = 0.008 *Amh*^-/-^, Student’s *t* test). **(C)** The *bars* illustrate the mean change in cell size after 20 days. In the female mice, the change in size was marginally significant (*p* = 0.048, two-way ANOVA of age and genotype). ♂, male; ♀, female.

In the 20-day-old mice, there was a significant effect of sex (*p* = 0.001, two-way ANOVA), *Amh* genotype (*p* = 0.008) and sex × genotype interaction (*p* = 0.022) on the number of calbindin^+ve^ neurons in their CALB-SDN. The wild-type 20-day-old pre-pubescent male mice had 47% more calbindin^+ve^ neurons in their CALB-SDN than their female littermates (Figures 2 and 3A), but the size and general appearance of the neurons were not overtly dimorphic (Figures 2 and 4A). This initial sex difference was absent in the *Amh*^*-/-*^ mice, with the *Amh*^*-/-*^ male mice containing numbers of neurons that were no different to the female mice (Figures 2 and 3A). The difference in the number of neurons between the *Amh*^*+/+*^ and *Amh*^*-/-*^ mice was highly statistically significant (*p* = 0.004, Student’s *t* test; Figure 3A). The size and appearance of the calbindin^+ve^ neurons in the *Amh*^*-/-*^ mice were indistinguishable from the *Amh*^*+/+*^ mice, for both males and females (Figures 2 and 4A).

The CALB-SDN underwent multiple changes between 20 days and adulthood. The number of calbindin^+ve^ neurons increased slightly in the *Amh*^*+/+*^ male mice (Figure 3B,C) and significantly decreased in the *Amh*^*+/+*^ female mice (Figure 3B,C). This caused the mean male-to-female ratio of neurons to increase from 1.47 (20 days) to 2.62 (adult). The male increase and female decrease in the number of calbindin^+ve^ neurons occurred in both the *Amh*^*+/+*^ and *Amh*^*-/-*^ mice (Figure 3C). Consequently, the proportion of dimorphism that is attributable to AMH decreased from approximately 100% at 20 days to 59% in the adult. In a two-way ANOVA test, the number of calbindin^+ve^ neurons were significantly different, with respect to sex (*p* < 0.001) and genotype (*p* = 0.002), with a significant sex × genotype interaction (*p* = 0.003).

The size of the neuronal soma also became dimorphic after 20 days of age, due to a slight hypertrophy in the males and a slightly larger atrophy in the females (Figure 4). The overall sex difference in the size of the calbindin^+ve^ neurons was, however, only 7%. As with neuronal number, the change in the size of the neuronal soma was similar in the *Amh*^*+/+*^ and *Amh*^*-/-*^ mice (Figure 4C).

The appearance of the calbindin^+ve^ neurons also became dimorphic after 20 days, with the intensity of the calbindin^+ve^ immunoreactivity being consistently stronger in the male than in the female mice (Figure 2). This was not due to variation in the immunohistochemical procedure as the brains were processed in groups of four, each of which contained one female and one male brain of each genotype. This difference was independent of the *Amh* genotype of the mice.

## Discussion

The control of the number of the calbindin^+ve^ neurons in the murine CALB-SDN is biphasic, with both a pre-pubertal phase and a phase that occurs after 20 days. These two phases appear to involve different sex-specific inducers, with the second phase involving changes in both female and male mice.

### Female puberty

In female mice, the number of calbindin^+ve^ neurons in the CALB-SDN decreased between 20 days and adulthood, indicating that some of the neurons in the pre-pubescent form of the nucleus either degenerated or differentiated into another cell type. This is consistent with archival descriptions of the SDN of the medial preoptic area in rats (herein referred to as SDN), which showed a decrease in the total volume of the nucleus in females between day 10 and adulthood [24]. A female-specific loss of neurons occurs during puberty in the rat prefrontal cortex [5], raising the possibility that pubescent loss of neurons may be a broad feature of female brain development. If so, then this would partially explain why the average size of the human female brain decreases by over 10% during puberty [25].

### Male puberty

The direction of pubescent change in the male CALB-SDN was opposite to that in the females, with the number of calbindin^+ve^ neurons increasing after 20 days. This is consistent with the recent study of Ahmed et al. demonstrating the existence of gonadal-dependent neurogenesis during puberty [4]. The magnitude of this increase was similar in the *Amh*^*+/+*^ and *Amh*^*-/-*^ mice, indicating that the pubescent increase in the size of the male CALB-SDN is independent of the pre-pubescent virilisation of the nucleus by AMH (see below). This notwithstanding, the number of calbindin^+ve^ neurons in the CALB-SDN of adult male *Amh*^*-/-*^ mice was still less than that of the *Amh*^*+/+*^ males, due to the different pre-pubescent development of the *Amh*^*-/-*^ and *Amh*^*+/+*^ mice. The functional significance of this will depend on whether the AMH-dependent and AMH-independent neurons in the CALB-SDN have similar or distinct roles.

The current study was not designed to examine the mechanism of the pubescent changes, only to detect whether a change occurs after 20 days. The data presented represent net changes. Consequently, neuronal loss, neurogenesis and/or change in cell type may be occurring in both sexes, with a female bias to the reduction in calbindin^+ve^ neurons and a male bias to the increase in calbindin^+ve^ neurons. It is also possible that some neurons change from a calbindin^+ve^ to a calbindin^-ve^ phenotype (or vice versa), although this is unlikely to be a complete explanation for the current data (Figures 3 and 4), given the rat observations noted above [4,24].

### Pre-pubertal ('childhood’) dimorphism and testosterone imprinting

High dimorphic levels of testosterone are only transiently present during development, with many features of the male brain being induced during this period. Some of the effects of testosterone during this period are mediated by the androgen receptor, whereas other effects are mediated by oestrogen receptors, after local aromatisation of testosterone [2]. The male features which develop between the perinatal and pubescent surges of testosterone are putatively generated by the prior exposure to testosterone. We refer to this as testosterone imprinting. As detailed below, the SDN of rats and the CALB-SDN in mice are exemplifiers of imprinting through the actions of aromatised testosterone. The dimorphism present in the CALB-SDN immediately before the onset of puberty (20 days) was absent in the *Amh*^*-/-*^ mice, indicating that most or all of this initial dimorphism is dependent on AMH. The blood levels of testosterone in *Amh*^*-/-*^ male mice are therefore of interest.

AMH is a natural repressor of Leydig cell formation [26] and a putative inhibitor of testosterone production [27], with pathological elevation of AMH levels leading to feminisation of mice, due to an apparent insufficiency of testosterone [28]. Conversely, *Amh*^-/-^ mice have an extensive Leydig cell hyperplasia [29,30], which, in isolation of other factors, should lead to increased levels of testosterone and increased sexual dimorphism. However, AMH also appears to negatively regulate the maturation of Leydig cells [30], with the results that testosterone levels in *Amh*^*-/-*^ male mice are within the normal male range [17,30,31], although data is not currently available for either foetal or neonatal mice. The potency of a hormone *in vivo* is regulated by binding proteins and other influences, with a biological readout of a hormone being particularly important for this reason. The male features of *Amh*^-/-^ mice that develop during either the foetal/neonatal or the pubertal surges of testosterone are quantitatively normal [17,29]. Hence, in our view, the complete absence of dimorphism in calbindin cell number in the 20-day-old mice is unlikely to be due to insufficient testosterone levels. This raises the possibility that AMH and the sex steroids dually regulate the CALB-SDN neurons (but see below). If so, then both AMH and testosterone may be needed to produce a male bias in the number of calbindin^+ve^ neurons.

Testosterone regulation of the neonatal SDN is postulated to involve local aromatisation of androgens to oestrogens [21,32,33]. Consequently, the putative actions of testosterone on this nucleus will be dependent on the expression of aromatase in the CALB-SDN as well as the plasma levels of testosterone. In this context, it will be important to determine whether the male bias in the expression of aromatase in the brain [34] is AMH dependent. If so, then feminisation of the CALB-SDN in the *Amh*^*-/-*^ male pups could be secondary to diminished conversion of testosterone to oestrogen.

Alternatively, the current results can be viewed as a challenge to the concept of testosterone imprinting. The non-dimorphic development of neurons are controlled by multiple factors, each of which acts at a particular stage of cell development (e.g. migration of the neuron, initial axon outgrowth, dendritic branching, etc.). All of these factors promote the survival of the neuron during development, but only the target-derived factor controls the number of neurons in the nucleus. When physiological regulators of neurons are injected at times when they are not normally present, then they mimic the action of the endogenous (natural) regulator. Consequently, if the physiological regulator of developmental cell death is added when the neuron is extending an axon, the regulator will promote axon outgrowth; conversely, if the physiological regulator of axon outgrowth is added during the period of cell death, then it will promote neuronal survival (see [35] for a discussion of this). For this reason, proof that a regulator is present at the time a cellular event is occurring is one of the most important criteria to prove the function of a non-dimorphic regulator of the brain (neurotrophic theory [35,36]).

The initial sex difference in the number of SDN neurons is created by a female bias in the extent of neuronal cell death [37,38]. The cell death in the SDN occurs after the neonatal surge of testosterone, during the period when the plasma levels of AMH but not testosterone are highly dimorphic (*cf.*[21,37] and [11,39]). The low adult ovarian production of AMH [13] is only beginning during this period [40]. The homologous nucleus in humans also develops when boys have high levels of AMH and only female-like levels of testosterone (*cf.*[41] and [9,42]). Hence, if neurotrophic theory is applicable, then this pattern of hormone secretion is consistent with AMH regulating the number of calbindin^+ve^ neurons of the CALB-SDN and is inconsistent with testosterone having this action.

All cytokine regulators of developing neurons leave a lasting imprint on neuronal networks, yet testosterone is the only regulator that is postulated to control neuronal development through an imprinting mechanism. The concept of testosterone imprinting was developed before the existence of gonadal protein hormones was known, when imprinting was the only apparent possibility for explaining the importance of the gonads. The proof of imprinting is predominantly based on experiments involving castration and the injection of non-physiological levels of the sex steroids into female mice. Castration removes testosterone, but it also removes AMH and other gonadal protein hormones (e.g. the inhibins [43-46]) that have dimorphic aspects to their secretion. Experiments based on castration are therefore open to multiple interpretations. Experiments involving the administration of the sex steroids are similarly open to debate for two reasons. First, the steroids have typically been used at high concentrations, without proof that the induced levels mirror the natural levels of testosterone in developing males. Of particular concern is whether steroids injected during the neonatal period persist into the subsequent days when developing males only have low levels of testosterone [11]. Second, both the testes and the ovaries produce the same protein hormones, but in different amounts and at different times in the life cycle. AMH, for example, is the most dimorphic of all the gonadal hormones during development, but is only minimally dimorphic in young adults [6,9-11]. Oestrogen and testosterone are paracrine regulators of the ovary [47], with oestrogen being a putative regulator of AMH production by ovarian granulosa cells [48]. The effect of non-physiological administration of the sex steroids on the secretion of protein hormones from the immature ovary (and testes) is largely unknown. In the absence of this information, any experiment involving a manipulation of one gonadal hormone has a degree of uncertainty because the effect on other hormones has not been fully characterised. This uncertainty extends in part to the current data. Hence, in our view, the concept of testosterone imprinting is not yet robustly proven.

This alternative view does not argue against the importance of the sex steroids. It simply suggests that the criteria used to identify the non-dimorphic regulators of the brain [35,36] are applicable to the study of the dimorphic regulators of the brain. If so, the available evidence points to testosterone regulating cellular events in the CALB-SDN before and after the AMH-mediated regulation of cell number. Non-dimorphic regulators of the brain have different actions on different types of neurons. Hence, the argument that AMH generates the male bias in neuron number in the CALB-SDN (this manuscript) and other neurons [14,17,49] is not an argument against testosterone having a similar action on neurons in populations where the control of cell number occurs during either the perinatal or the pubescent surges of testosterone.

## Conclusions

The control of neuron number in the CALB-SDN appears to be biphasic, with the different phases subject to different regulation. One interpretation of this data is that organisation and activation events occur early in development and during puberty, with the testicular influence being continuous through direct non-imprinting effects of protein and steroid hormones, acting at different times.

## Competing interests

The authors declare that they have no competing interests.

## Authors’ contributions

Both authors designed the study, interpreted the results and drafted the manuscript. WW collected the data. Both authors read and approved the final manuscript.
